# Screening for Antiviral Activities of Isolated Compounds from Essential Oils

**DOI:** 10.1093/ecam/nep187

**Published:** 2011-02-14

**Authors:** Akram Astani, Jürgen Reichling, Paul Schnitzler

**Affiliations:** ^1^Department of Infectious Diseases, Virology, University of Heidelberg, 69120 Heidelberg, Germany; ^2^Yazd Shahid Sadoghi University of Medical Science, Safaieh, Yazd, Iran; ^3^Department of Biology, Institute of Pharmacy and Molecular Biotechnology, University of Heidelberg, Germany

## Abstract

Essential oil of star anise as well as phenylpropanoids and sesquiterpenes, for example, trans-anethole, eugenol, *β*-eudesmol, farnesol, *β*-caryophyllene and *β*-caryophyllene oxide, which are present in many essential oils, were examined for their antiviral activity against herpes simplex virus type 1 (HSV-1) *in vitro*. Antiviral activity was analyzed by plaque reduction assays and mode of antiviral action was determined by addition of the drugs to uninfected cells, to the virus prior to infection or to herpesvirus-infected cells. Star anise oil reduced viral infectivity by >99%, phenylpropanoids inhibited HSV infectivity by about 60–80% and sesquiterpenes suppressed herpes virus infection by 40–98%. Both, star anise essential oil and all isolated compounds exhibited anti-HSV-1 activity by direct inactivation of free virus particles in viral suspension assays. All tested drugs interacted in a dose-dependent manner with herpesvirus particles, thereby inactivating viral infectivity. Star anise oil, rich in trans-anethole, revealed a high selectivity index of 160 against HSV, whereas among the isolated compounds only *β*-caryophyllene displayed a high selectivity index of 140. The presence of *β*-caryophyllene in many essential oils might contribute strongly to their antiviral ability. These results indicate that phenylpropanoids and sesquiterpenes present in essential oils contribute to their antiviral activity against HSV.

## 1. Introduction

Herpes simplex virus type 1 (HSV-1) is an important pathogen for humans, and discovery of novel effective antiherpetic drugs without adverse effects is of great interest. The primary symptoms of herpes infection include a prodromal flu-like syndrome with fever, headache, malaise, diffuse myalgias, followed by local symptoms consisting of itching and painful papules. Gingivostomatitis and pharyngitis are the most frequent clinical manifestations of first episodes of HSV-1 infection. After establishing latency, HSV can reactivate, causing frequent recurrent infections in some patients, whereas most people experience few recurrences [1]. The clinical manifestation of the disease exhibits different severity in immunocompetent patients and in addition some patients always encounter recurrent attacks [2]. However in immunocompromised patients and neonates, herpetic infections can cause serious systemic illnesses. Recurrent herpes labialis is the most frequent clinical manifestation of reactivated HSV-1 infection.

A very effective treatment for HSV is available since the introduction of acyclovir in the 1970s and it is still the most commonly used chemotherapy [3]. This antiviral agent can be used to shorten the course and decrease the severity of these clinical symptoms and may suppress the virus itself [2]. Antiviral agents licensed currently for the treatment of herpesvirus infections include acyclovir and derivatives, foscarnet and cidofovir, all of which inhibit herpesvirus DNA polymerases [4]. Some of these antiviral agents might produce toxic side-effects. In addition, the emergence of virus strains resistant to commonly used anti-herpesvirus drugs is of importance, particularly in immunocompromised patients [5–7]. The development of viral resistance toward antiviral agents enhances the need for new effective compounds against viral infections. Thus, new antiviral agents exhibiting different mechanisms of action are urgently needed.

Medicinal plants produce a variety of chemical constituents with the potential to inhibit viral replication and compounds from natural sources are of interest as possible sources to control viral infection. These plants have been widely used to treat a variety of infectious and non-infectious diseases and represent an abundant source of new bioactive secondary metabolites. Thus plants continue to be a major source of new lead compounds. Besides small molecules from medicinal chemistry, natural products are still major sources of innovative therapeutic agents for various conditions, including infectious diseases. In recent years there has been an increasing interest in the use of natural substances, and some questions concerning the safety of synthetic compounds have encouraged more detailed studies of plant resources. Essential oils, odors and volatile products of plant secondary metabolism, have a wide application in folk medicine as well as in fragrance industries. Essential oils are complex natural mixtures of volatile secondary metabolites, isolated from plants by hydro- or steam-distillation. The main constituents of essential oils, for example, monoterpenes and sesquiterpenes and phenylpropanoids including carbohydrates, alcohols, ethers, aldehydes and ketones, are responsible for the fragrant and biological properties of aromatic and medicinal plants [8]. Various essential oils and their components possess pharmacological effects, demonstrating antiinflammatory, antioxidant and anticancerogenic properties [9–11].

Antiherpes screening experiments on medicinal plant extracts and plant-derived secondary metabolites have been reported [8, 12]. Antibacterial, antifungal, immunomodulatory, antiinflammatory and antirheumatic activities have been described for essential oils [8, 13–21]. The antiherpes activity of several essential oils of different plant sources as well as of some constituents of essential oils had been demonstrated previously [22–25]. The application of tea tree oil, the essential oil of *Melaleuca alternifolia*, for the treatment of recurrent herpes labialis has been reported recently [26]. The antiherpes activity of eucalyptus oil, Australian tea tree oil [27], thyme oil [28] and manuka oil [14] has previously been published. Other medicinal plant extracts of traditional Thai medicinal plants showed a pronounced antibacterial activity against methicillin-resistant *Staphylococcus aureus* [29]. Christoph et al. [30, 31] performed a comparative study on the *in-vitro* antimicrobial activity of tea tree oil with special reference to the activities of *β*-triketones. Some phenylpropanes [32, 33], triterpenes [34] and sesquiterpenes [35–37] had been tested for their antiviral activity against different herpesviruses and rhinovirus. However, sesquiterpenes as important constituents of essential oils, have not been analyzed systematically for their antiviral potential. Only few reports describe the inhibition of viral replication by sesquiterpenes, for example, triptofordin C-2 [35]. Thus only limited information about sesquiterpenes concerning the inhibition of the viral replication cycle and their mode of antiviral action is presently available.

The aim of the present study is the evaluation of the antiviral activity of selected sesquiterpenes, important constituents of essential oils, against HSV-1 and the mode of antiviral action of these sesquiterpenes during the viral multiplication cycle.

## 2. Methods

### 2.1. Star Anise Oil, Phenylpropanoids, Sesquiterpenes, Acyclovir

**Figure 1:**
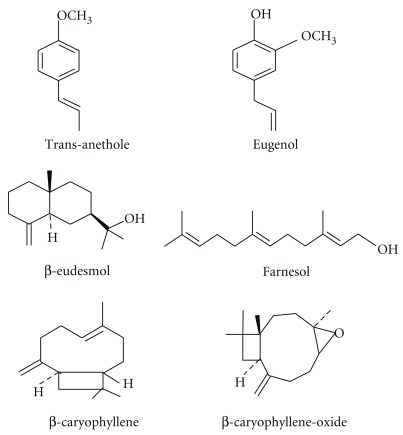
Structural formulas of phenylpropanoids and sesquiterpenes.

Star anise essential oil met the standard demands of current pharmacopoeias and literature data [38] and was obtained from Caelo (Hilden, Germany). This essential oil is rich in trans-anethole and consists of about 80% of this phenylpropanoid [39]. Trans-anethole, eugenol, *β*-caryophyllene were purchased from Roth GmbH (Karlsruhe, Germany), and *β*-eudesmol, farnesol and *β*-caryophyllene oxide were purchased from Sigma-Aldrich Chemie GmbH (Taufkirchen, Germany). All phenylpropanoids and sesquiterpenes met high purity standards. High contents of eugenol in clove oil and trans-anethole in star anise oil have been reported previously [8]. Structural formulas of these selected phenylpropanoids and sesquiterpenes constituents are presented in Figure 1. Star anise oil and selected compounds were dissolved in ethanol and further diluted in medium for cell culture experiments, always resulting in an ethanol concentration <1%, which has no effect on cells and viruses [28]. Acyclovir, a commonly used anti-HSV synthetic drug, was purchased from GlaxoSmithKline (Bad Oldesloe, Germany), dissolved in sterile water and applied as reference compound.

### 2.2. Cell Culture and HSV-1

RC-37 cells (African green monkey kidney cells) were grown in monolayer culture with Dulbecco's modified Eagle's medium (DMEM; Gibco, Karlsruhe, Germany) supplemented with 5% fetal calf serum (FCS; Gibco, Karlruhe, Germany), 100 U mL^−1^ penicillin and 100 *μ*g mL^−1^ streptomycin (both Gibco, Karlsruhe, Germany). The monolayers were removed from their plastic surfaces and serially passaged whenever they became confluent. Cells were plated onto 96-well and 6-well culture plates for cytotoxicity and antiviral assays, respectively, and propagated at 37°C in an atmosphere of 5% CO_2_. HSV-1 strain KOS was used for all experiments. Viruses were routinely grown on RC-37 cells and virus stock cultures were prepared from supernatants of infected cells and stored at −80°C. Infectivity titers were determined by a standard plaque assay on confluent RC-37 cells [40].

### 2.3. Cytotoxicity Assay

The effect of star anise oil, phenylpropanoids and sesquiterpenes on the proliferation of RC-37 cells was determined in 96-well tissue culture plates at an initial density of 1 × 10^5^ cells mL^−1^. For cytotoxicity assays, cells were seeded onto 96-well plates and incubated for 24 h at 37°C. The medium was removed and fresh DMEM containing the appropriate dilution of the essential oil or compounds was added onto subconfluent cells in eight replicates for each concentration of the drugs. Wells containing medium with 1% ethanol but no drug were also included on each plate as controls. After 3 days of incubation, the growth medium was removed and viability of the drug-treated cells was determined in a standard neutral red assay [41]. Neutral red dye uptake was determined by measuring the optical density (OD) of the eluted neutral red at 540 nm in a spectrophotometer. The mean OD of the cell-control wells was assigned a value of 100%. The cytotoxic concentration of the drug that reduced viable cell number by 50% (TC_50_) was determined from dose-response curves. Additionally the maximum noncytotoxic concentration of each drug was determined.

### 2.4. Dose-Response Assays

The antiviral activity of star anise oil, phenylpropanoids and sesquiterpenes was assayed by plaque reduction assay. A virus suspension of HSV-1 containing 2 × 10^3^ plaque forming units (pfu mL^−1^) was incubated with an equal volume of DMEM or various concentrations of star anise oil or phenylpropanoids and sesquiterpenes for 1 h at room-temperature, then virus was allowed to adsorb to the cells for 1 h at 37°C. The residual inoculum was replaced by medium containing 0.5% methylcellulose. After incubation for 3 days at 37°C, monolayers were fixed with 10% formalin. The cultures were stained with 1% crystal violet and subsequently the plaques were counted. Each concentration was performed in three replicates, virus-infected cells in wells containing medium with 1% ethanol but no drug were also included on each plate as controls. Inhibitory concentration (IC_50_) was expressed as antiviral activity, which inhibited plaque numbers by 50% compared with untreated control and was determined from dose-response curves.

### 2.5. Time of Addition Studies

Star anise oil, phenylpropanoids or sesquiterpenes were added to the cells before, during and after virus infection. The maximum noncytotoxic concentration was always used to evaluate the mode of antiviral action. Cell monolayers were pretreated with drugs prior to inoculation with virus by adding the oil or compounds to the medium followed by incubation for 1 h at 37°C. For pretreatment of HSV with drugs, about 2 × 10^3^ pfu of HSV were incubated in medium containing the drugs for 1 h at room-temperature prior to infection of RC-37 cells. The effect of essential oil or components against HSV was also tested during the replication period by addition of drugs after cell infection to the overlay medium, as typically performed in antiviral susceptibility studies. Each assay was run in three replicates. Plaque reduction assays were carried out as described above and number of plaques of drug-treated cells and viruses were compared with untreated controls. Wells containing medium with 1% ethanol but no drug were also included on each plate as controls.

### 2.6. Statistical Analysis

The selectivity index (SI) was determined by the ratio of TC_50_ to IC_50._ All experiments were performed in triplicate, and three independent experiments were conducted. Data were presented as mean ± SD and *t*-test was used to evaluate the difference between the test and control. A *P*-value of < .05 was considered statistically significant.

## 3. Results

### 3.1. Cytotoxicity of Essential Oil Compounds

Star anise oil and six selected phenylpropanoids and sesquiterpenes (Figure 1) were serially diluted in ethanol and added to cell culture medium to examine the effect on the growth and viability of tissue culture cells, always resulting in an ethanol concentration <1%, which had no effect on cells and viruses. After 3 days of incubation, cell viability of RC-37 cells was determined with the neutral red assay (Table 1). The maximum noncytotoxic concentrations of these drugs were determined between 9 *μ*g mL^−1^ for *β*-caryophyllene oxide and *β*-eudesmol and 100 *μ*g mL^−1^ for star anise oil, the drug which revealed the lowest cytotoxicity. Similar results were found for TC_50_ values, for example, 18 *μ*g mL^−1^ for *β*-caryophyllene oxide and 160 *μ*g mL^−1^ for star anise oil.

### 3.2. Antiviral Activity of Essential Oil Compounds

**Figure 2:**
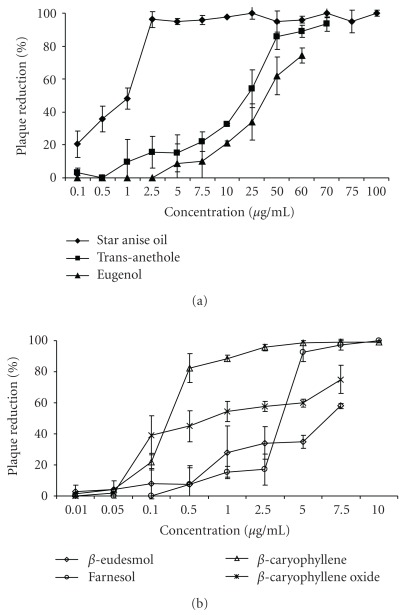
Antiviral activity of serial dilutions of (a) star anise oil, phenylpropanoids and (b) sesquiterpenes against HSV-1 in viral suspension assays. Diluted drugs were tested up to the maximum noncytotoxic concentration. Number of virus plaques was determined 3 days after infection and compared to untreated control. Results are presented as percentage of plaque reduction, experiments were repeated independently and data are the mean of three experiments ± SD.

The potential antiviral effect of star anise essential oil and some selected components from different essential oils was determined against HSV-1 *in vitro*. HSV-1 was incubated for 1 h at room-temperature with various concentrations of star anise oil, trans-anethole, eugenol, *β*-eudesmol, farnesol, *β*-caryophyllene and *β*-caryophyllene oxide. In all assays untreated virus-infected cells were used as a control. Subsequently, aliquots of each dilution were incubated with RC-37 cells for 1 h, then the cells were washed and overlaid with drug-free medium and incubated for 3 days at 37°C. The 50% inhibitory concentrations (IC_50_) for HSV-1 were determined in a wide range between 0.25 *μ*g mL^−1^ for *β*-caryophylle and 35 *μ*g mL^−1^ for eugenol (Table 1). The results are presented in Figures 2(a) and 2(b) as virus reduction and represent the average of three independent experiments. In plaque reduction assays, star anise oil and compounds exhibited a concentration-dependent antiviral effect, star anise oil with a SI of 160 was the most effective drug and slightly superior to *β*-caryophyllene with a SI of 140. SIs for tested drugs against HSV were calculated as the TC_50_/IC_50_ ratio. The essential oil of star anise was able to suppress viral multiplication by >99% (Figure 2(a)). Out of six tested compounds, only trans-anethole, *β*-caryophyllene and farnesol suppressed herpesvirus infectivity by >90% at the maximum noncytotoxic concentration of these drugs.

### 3.3. Mechanism of Antiviral Action

**Figure 3:**
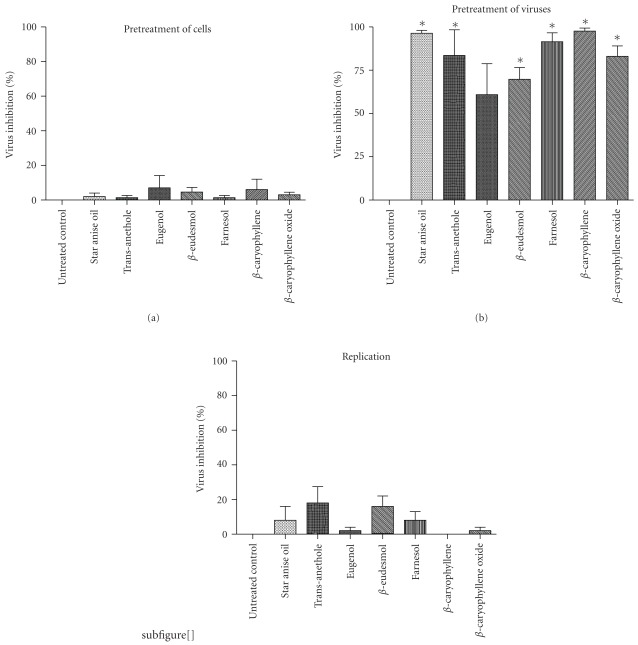
Antiviral activity of star anise essential oil and different compounds of essential oils against HSV in time of addition assays. (a) Pretreatment of cells with drugs, (b) pretreatment of virus with drugs and (c) addition of drugs during intracellular replication of HSV. Number of virus plaques was determined 3 days after infection and compared to untreated control. Results are presented as percentage of plaque reduction and are the mean of three independent experiments ± SD and statistically significant results are marked with asterisk.

For investigation of the inhibitory effect on HSV in detail, all drugs were added at different stages during viral infection. For comparison, all untreated controls contained the same concentration of ethanol as the drug-treated viruses, in order to exclude any influence of ethanol. When host cells were pretreated with drugs prior to infection, none of the tested drugs showed statistically significant effects (*P* = 0.2–0.4) on viral infection (Figure 3(a)). On the other hand, pretreatment of HSV-1 with star anise oil, phenylpropanoids or sesquiterpenes prior to infection inhibited herpesvirus infectivity. At maximum noncytotoxic concentrations of the tested drugs, infectivity was reduced by >99% for star anise oil followed by 98% reduction for *β*-caryophyllene (Figure 3(b)). All other constituents of essential oils revealed a plaque reduction of HSV between about 60 and 90%. However, the antiviral effect of eugenol was not statistically significant (*P* = .07). Acyclovir showed the highest antiviral activity when added during the replication period with inhibition of the viral replication of >99% (data not shown). This drug inhibits specifically the viral DNA polymerase during the replication cycle when new viral DNA is synthesized. In contrast, when the oil or compounds were added to the overlay medium after penetration of the viruses into the host cells, plaque formation was not significantly (*P* = 0.2–0.4) reduced (Figure 2.6).

## 4. Discussion

The pharmaceutical industry is increasingly targeting medicinal plants with the aim of identifying lead compounds, focusing particularly on suitable alternative antiviral agents. Topical treatment of herpes labialis infection is standard, for the most part carried out not only with acyclovir creams, but also with phytopharmaceuticals containing sage or lemon balm extracts [42–44]. Both plant extracts were shown to be significantly superior to placebo and equivalent to acyclovir [43]. Our previous *in-vitro* experiments revealed similar results for essential oils from eucalyptus, tea tree and thyme [18, 27, 45, 46]. In the present study, the inhibitory effect of star anise oil against HSV infection was compared with the antiviral potential of phenylpropanoid and sesquiterpene compounds. Experiments to assess the cytotoxicity of essential oils and monoterpenes for cultured eukaryotic cells indicate a moderate toxic behavior in cell cultures according to Halle and Göres [47]. Star anise essential oil and most compounds exhibited high levels of antiviral activity against HSV-1 in viral suspension tests. At maximum noncytotoxic concentrations plaque formation was significantly reduced by >99% for star anise oil, phenylpropanoids and sesquiterpenes were able to suppress viral infection by 60–80% and 40–98%, respectively.

**Figure 4:**
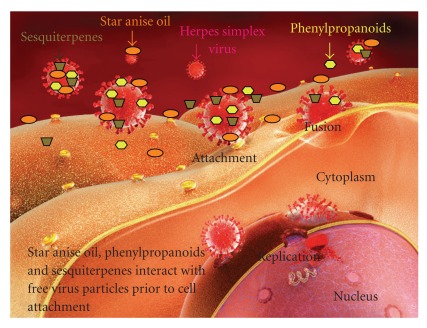
Star anise oil, phenylpropanoids and sesquiterpenes exhibited antiviral activity by direct interaction with free virus particles. Pretreatment of cells with the drugs had no effect on viral infectivity, neither during intracellular viral replication.

The mode of antiviral action was determined in time of addition assays. Pretreatment of the cells with these drugs had no effect on the production of infectious virus and plaque formation. The same results were found when star anise oil or compounds were added during the replication period of the infection cycle. However, high antiviral activity was observed for star anise oil, trans-anethole, farnesol and *β*-caryophyllene when herpesvirus was incubated with these drugs prior to host cell infection (Figure 4). These results suggest that these drugs directly inactivate herpes virus and might interfere with virion envelope structures or mask viral structures that are necessary for adsorption or entry into host cells. A virucidal activity of *Melaleuca armillaris* essential oil has been reported recently [27] and dissolution of the HSV envelope by treatment with oregano essential oil has been described [48]. Thus different mechanisms of antiviral activity of different essential oils and compounds of essential oils seem to be present. De Logu et al. [12] reported an inactivation of herpesviruses and prevention of cell-to-cell spread by *Santolina insularis* essential oil. However, no antiviral effect was observed during the intracellular replication phase, which is in accordance to our results and other essential oils [46]. Adenovirus, a virus without envelope, was not affected by eucalyptus essential oil due to the lack of a viral envelope [21]. Sesquiterpenes, for example, triptofordin C-2 and sesquiterpene coumarins inhibit cytomegalovirus [35], severe acute respiratory syndrome coronavirus [49] and rhinovirus [37]. Pusztai et al. [36] reported a specific inhibition of the CMV immediate early gene expression, whereas other sesquiterpenes are moderately virucidal against different enveloped viruses, for example, HSV, cytomegalovirus, measles virus and influenza virus [35]. Eugenol, a phenylpropane that represents about 75% v/v in clove essential oil, delayed the development of herpesvirus-induced keratitis in the mouse model [32] and inactivated HSV directly [39]. Direct inactivation of virus particles by eugenol represents the same antiviral mechanism as examined for the constituents in this study. Isoborneol, a monoterpene and a component of several plant essential oils, showed virucidal activity against HSV-1 and specifically inhibited glycosylation of viral proteins [50]. The application of cineole protects mice against infection with HSV-2 [22]. Since essential oils are able to inhibit acyclovir-resistant HSV-1 isolates [28], the mechanism of interaction between these compounds and acyclovir with HSV must be different. Acyclovir inhibits virus replication by interference with the DNA polymerase inside the cell, whereas star anise oil, phenylpropanoids and sesquiterpenes probably inactivate HSV before it enters the cell. Viral resistance to acyclovir represents a particular problem, the prevalence of resistance in acyclovir-treated immunocompromised individuals is ~4–7% [51, 52]. Therefore other antiherpetic agents that are effective for viral mutants resistant to current antiviral agents are of great interest for topical treatment. The application of tea tree oil, the essential oil of *Melaleuca alternifolia*, for the treatment of recurrent herpes labialis has been reported recently [26, 53].

The complex mixture of the essential oil revealed a higher antiviral activity and SI of 160, whereas single constituents revealed lower selectivity indices. However *β*-caryophyllene revealed a SI of 140, which is in the same range as the index value of 160 for star anise oil and is found in many different essential oils from different plant families. Thus *β*-caryophyllene might be one of the dominant antiviral agents in different essential oils. The antiviral potential of the test compounds in our study can be traced back to their structural features. The sesquiterpene hydrocarbon *β*-caryophyllene is the most active antiviral compound with an IC_50_ of 0.25 *μ*g mL^−1^. The introduction of either an epoxide or hydroxyl function into the sesquiterpene backbone led to a moderate decrease in its antiviral effect. Cos et al. [54] recommended IC_50_ values for promising natural products against infectious diseases, for example, for extracts <100 *μ*g mL^−1^. Star anise essential oil in our study revealed an IC_50_ values of 1 *μ*g mL^−1^ and is far below the recommended cutoff and presents a promising antiinfective agent according to this recommendation.

In conclusion, medicinal and aromatic plants are widely used today in modern phytotherapy [55]. The essential oils and their components are known to be active against a wide variety of microorganisms [56]. Phenylpropanoids and sesquiterpenes present in essential oils contribute to their antiviral activity. Drugs with a high SI are preferable for antiviral treatment in patients, thus star anise oil as a complex mixture and *β*-caryophyllene as single constituent might be applied as topical therapeutic agents in the treatment of recurrent herpes infection.

## Funding

Grant from Iranian Ministry of Health (to A. Astani).

## Figures and Tables

**Table 1:** Selectivity indices of anise oil and selected phenylpropanoids and sesquiterpenes against HSV-1^(a)^.

Essential oil/compound	Max. non-cytotoxic conc. (*μ*g mL^−1^) ± SD	TC_50_ (*μ*g mL^−1^) ± SD	IC_50_ (*μ*g mL^−1^) ± SD	SI
Star anise oil	100 ± 8.0	160 ± 30.7	1 ± 0.1	160
Trans-anethole	70 ± 3.0	100 ± 6.4	20 ± 1.1	5
Eugenol	60 ± 11.1	85 ± 8.1	35 ± 6.2	2.4
*β*-Eudesmol	9 ± 1.3	35 ± 5.4	6 ± 0.3	5.8
Farnesol	10 ± 0.4	40 ± 3.7	3.5 ± 0.1	11.4
*β*-Caryophyllene	10 ± 0.1	35 ± 2.3	0.25 ± 0.0	140
*β*-Caryophyllene oxide	9 ± 1.1	18 ± 1.2	0.7 ± 0.1	25.7

^(a)^Experiments were repeated independently and data presented are the mean of three experiments.
